# Auxeticity of Concentric Auxetic-Conventional Foam Rods with High Modulus Interface Adhesive

**DOI:** 10.3390/ma11020223

**Published:** 2018-01-31

**Authors:** Teik-Cheng Lim

**Affiliations:** School of Science and Technology, Singapore University of Social Sciences, Singapore 599494, Singapore; tclim@suss.edu.sg; Tel.: +65-9137-9352

**Keywords:** adhesive, auxetic, concentric cylinders, foam, torsion, tri-layered

## Abstract

While the rule of mixture is applicable for addressing the overall Poisson’s ratio of a concentrically aligned bi-layered rod under longitudinal loading, the same cannot be said for this rod under torsional loading due to the higher extent of deformation in the rod material further away from the torsional axis. In addition, the use of adhesives for attaching the solid inner rod to the hollow outer rod introduces an intermediate layer, thereby resulting in a tri-layered concentric rod if the adhesive layer is uniformly distributed. This paper investigates the effect of the adhesive properties on the overall auxeticity of a rod consisting of two concentrically aligned cylindrical isotropic foams with Poisson’s ratio of opposite signs under torsional loads. An indirect way for obtaining Poisson’s ratio of a concentrically tri-layered rod was obtained using a mechanics of materials approach. Results show that the auxeticity of such rods is influenced by the adhesive’s stiffness, Poisson’s ratio, thickness, and radius from the torsional axis.

## 1. Introduction

In the analysis of negative materials, the term “conventional materials” is typically adopted when referring to materials with properties that are normally taken for granted, such as positive Poisson’s ratio, positive thermal expansivity, and positive stiffness. For papers that deal with auxetic materials, “conventional” materials are those that exhibit a positive Poisson’s ratio. The problem of torsion of cylindrical rods made from conventional materials is well established and has been a subject of extensive research. The theory of generalized torsion was first worked out by Voigt and the rigorous theory of pure torsion was developed by Saint–Venant. There are many classical works on the theory of pure torsion (e.g., [1,2,3,4]). Tsukrov and Drach [5] gave explicit analytical expressions for displacement and stress fields in a multilayered composite cylinder with cylindrically orthotropic layers subjected to homogeneous boundary conditions. The solutions are derived under the assumption of perfect bonding between layers. Torsion of laminated cylindrical shells with adhesive interlayers was investigated by Maksymuk and Shcherbina [6]. Based on refined equations of the Timoshenko-type shell theory, the contact stresses in torsion of a two-layer cylindrical shell with an adhesive interlayer were numerically studied. The effect of the geometric and physical-mechanical parameters of the load-carrying layers and the adhesive interlayer of the shell on the distribution of the interlaminar tangential stress was analyzed as well. The magnetoelectric (ME) effect in a piezoelectric/piezomagnetic (PE/PM) composite cylinder, which is induced by a torsional deformation, was investigated by Huang and Zhang [7]. Both the PE and PM layers are circumferentially polarized. For a specific applied magnetic field, the displacement component in the PM layer was analytically presented. Levin et al. [8] considered a static problem of torsion of a cylinder composed of incompressible, nonlinear elastic materials at large deformations. The cylinder contained a central, round, cylindrical inclusion that was initially twisted and stretched (or compressed) along the axis and fastened to a strainless, external, hollow cylinder.

Since 1987, when negative Poisson’s ratio foams were developed by Lakes [9,10], it is known that materials and structures showing a negative Poisson’s ratio do exist in nature. Popereka and Balagurov [11] presented ferromagnetic films having a negative Poisson ratio; Milstein and Huang [12] confirmed the existence of a negative Poisson ratio in face-centered cubic (fcc) crystals. Simple mechanical [13,14,15] and thermodynamic [16] models, which show auxetic behavior, were found in the 80s of the 20th century; the latter was studied in the form of hexagonal molecules in two-dimensional (2D) lattice [17,18]. The counterintuitive nature of auxetic materials avails much potential as safer fasteners [19], improved arterial prostheses [20], artificial intervertebral discs [21], drug eluting stents [22] and other prostheses [23], more comfortable cushions [24,25], highly tunable molecular sieves [26,27,28,29], antivibration gloves [30], novel textiles [31], better material for diabetic shoes [32], safer pressure vessels [33] and other thin-walled structures [34], and a better performing crash/shock absorber [35,36]. It has also been found that novel properties, distinct from conventional or auxetic materials, arise when a composite structure is made from both conventional and auxetic materials. In addition to a recent comprehensive survey of auxetic materials and structures [37], the reader is also referred to a recent focus issue on “auxetics and other systems of anomalous characteristics” [38,39,40,41,42], as well as papers in this special issue [43,44,45,46,47,48].

Interlacing effects from the auxetic and conventional materials have been shown to give interesting properties in bi-layer [49,50], tri-layer [51,52], and multi-layered systems [53,54,55,56,57,58,59,60], and continuous unidirectional fiber composites [61]. One category of auxetic structural element is that of rods possessing circular or elliptical cross-sections with auxetic and conventional materials alternated in concentric [62,63], angular [64], and helical [65] directions. The earlier approach [62] assumes that (a) the interface between both the auxetic and conventional phases has no thickness and that (b) the bonding between both phases is perfect; due to finite element approximation used in the numerical model of the two-phase composites [63,64,65], an “interface layer” with thickness is present instead of the perfect bond between the material. In practice, adhesion is required between both phases such that the interface between both phases is replaced by an interphase and that the interphase has its own material properties and thickness. This paper attempts to model, by an analytical approach, a single tri-layered cylindrical rod consisting of a solid inner foam rod adhered to a hollow outer foam rod, with significant adhesive modulus as the interface of the two foam rods.

The overall auxeticity of a concentric rod whereby the core and shell possess Poisson’s ratio of opposite signs is influenced by the mode of loading [62]. It is obvious that during axial loading both the core and shell experience equal longitudinal strain but during torsional loading, the shell undergoes greater shear strain than the core. In this concentrically bi-layered rod system, the auxeticity during axial and torsional loading modes changes at different rates with respect to the ratio of the inner-to-outer diameters. This results in a range of inner-to-outer diameter ratios whereby the concentric rod exhibits overall Poisson’s ratio of different signs depending on the loading mode. However, the assumption of a bi-layered concentric foam system is no longer valid when a layer of high modulus adhesive exists between the two concentric rods. The adhesive layer can be thought of as an intermediate layer that fills the gap between the two concentric cylinders. Even in the case where the surfaces of the inner and outer cylinders are in contact with each other, the slight seepage of adhesive fluid into the foam before its solidification gives rise to a high modulus intermediate layer. The effect of the adhesive’s elastic properties under torsional load on the concentric foam rods of opposing Poisson’s ratio signs, but with comparable Young’s modulus, is investigated in this paper as a tri-layered concentric rod system.

## 2. Theory and Formulation

**Figure 1:**
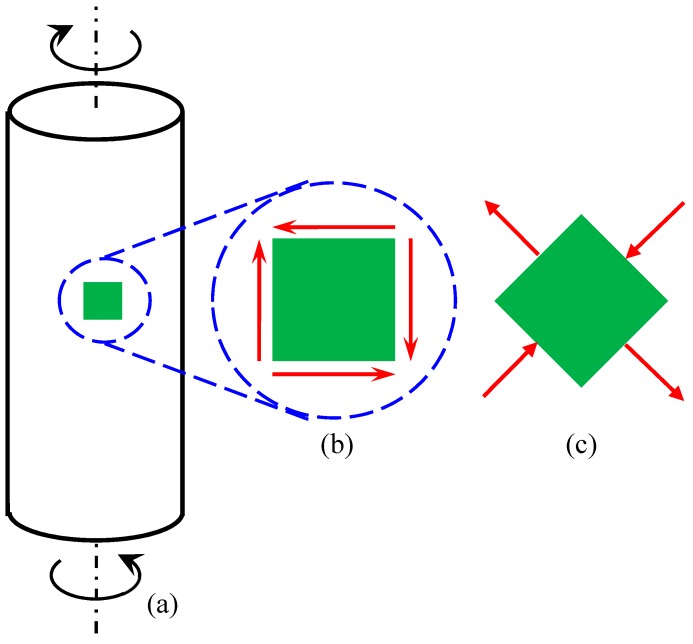
Schematics for (**a**) a rod under torsion, (**b**) an elemental volume under pure shearing, and (**c**) an equivalent stress state at 45° showing in-plane principal stresses. Red arrows indicate stress directions.

Unlike uniaxial loading whereby the diameter changes for non-zero Poisson’s ratio, there is no salient change in the rod diameter under torsional load. With reference to Figure 1, a component of radial increase due to one principal strain is canceled by a component of radial decrease due to another principal strain in the same plane. Hence, there is no change in the rod diameter under torsion. Nevertheless, it is obvious that the auxeticity of the rod must be related to that of the material that constitutes the rod. 

### 2.1. Single Solid Rods

Since an observation on rod diameter yields no change regardless of the rod auxeticity, we herein consider the rod auxeticity—under torsional loading—in terms of the moduli ratio *G*/*E* (or *E*/*G*) using the elastic relation:

(1) $$G=\frac{E}{2(1+v)}.$$

By virtue of Equation (1), the moduli ratio *G*/*E*→∞ (or *E*/*G*→0) as *v*→−1, and that *G*/*E*→1/3 (or *E*/*G*→3) as *v*→1/2. Hence, the auxeticity of a material can be inferred from the moduli ratio as an alternative to the usual way of measuring the change in dimension during axial loading. For a single solid rod of diameter *D* and length *L* undergoing torsion *T*, the angular twist *φ* is given as

(2) $$\varphi =\frac{TL}{GJ}$$

whereby the polar moment area of the circular cross-section *J* is

(3) $$J=\frac{\pi }{32}D^{4}.$$

Substituting Equations (1) and (3) into Equation (2), we have Poisson’s ratio of the rod:

(4) $$v=\frac{D^{4}E\pi \varphi }{64TL}-1$$

or, for convenient comparison with subsequent sub-sections, we write

(5) $$v_{Single}={\left(\frac{64TL}{D^{4}E\pi \varphi }\right)}^{-1}-1.$$

### 2.2. Bi-Layered Concentric Rods

**Figure 2:**
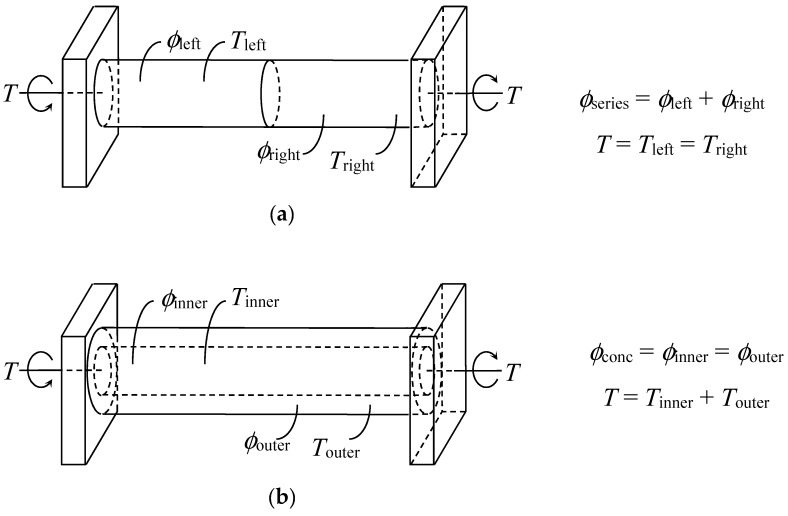
Comparison between two rods in (**a**) series and (**b**) concentric arrangement.

As opposed to summative angular twist and common transmitted torsional load for two rods arranged in series, the case of two concentrically arranged rods is governed by a common angular twist with summative torsional loads as depicted in Figure 2a,b, respectively.

Hence, the common angular twist for the inner and outer rods

(6) $$\varphi_{i}=\varphi_{o}$$

and the summative torsional load

(7) $$T=T_{i}+T_{o}=\frac{\varphi_{i}G_{i}J_{i}}{L}+\frac{\varphi_{o}G_{o}J_{o}}{L}$$

give

(8) $$\frac{TL}{\varphi }=G_{i}J_{i}+G_{o}J_{o}.$$

Substituting Equations (1) and (3) into Equation (8) leads to

(9) $$\frac{64TL}{D_{o}{}^{4}E_{o}\pi \varphi }=\frac{\frac{E_{i}}{E_{o}}{\left(\frac{D_{i}}{D_{o}}\right)}^{4}}{1+v_{i}}+\frac{1-{\left(\frac{D_{i}}{D_{o}}\right)}^{4}}{1+v_{o}}.$$

It can be easily seen that Equation (9) can be reduced to Equation (5) under certain special cases; substituting *D_i_* = 0 (i.e., *E* = *E_o_* for the entire rod) into Equation (9) leads to

(10) $$\frac{64TL}{D_{o}{}^{4}E_{o}\pi \varphi }=\frac{1}{1+v_{o}}$$

or

(11) $$v_{o}={\left(\frac{64TL}{D_{o}{}^{4}E_{o}\pi \varphi }\right)}^{-1}-1$$

while the substitution of *D_i_* = *D_o_* (i.e., *E* = *E_i_* for the whole rod) into Equation (9) gives

(12) $$\frac{64TL}{D_{o}{}^{4}E_{i}\pi \varphi }=\frac{1}{1+v_{i}}$$

or

(13) $$v_{i}={\left(\frac{64TL}{D_{o}{}^{4}E_{i}\pi \varphi }\right)}^{-1}-1.$$

Hence, by virtue of Equation (5) and neglecting the adhesive layer, the effective Poisson’s ratio for two perfectly bonded concentric cylinders under torsional loading mode is

(14) $$v_{Bi-layer}={\left(\frac{64TL}{D_{o}^{4}E_{o}\pi \varphi }\right)}^{-1}-1$$

where the term in parenthesis is given by Equation (9) in the case of a bi-layered concentric rod. However, to prevent unnecessary error, caution must be taken when using Equation (9) under some limiting conditions. For example, it is obvious that *E* = *E_i_* and *v* = *v_i_* for the entire rod if we let *D_i_* = *D_o_*; however, substituting *D_i_*/*D_o_* = 1 and *E_i_*/*E_o_*→∞ into Equation (9) results in *v* = −1 instead of *v* = *v_i_*. The source of this error can be traced by considering the fact that the substitution of *D_i_*/*D_o_* = 1 into Equation (9) implies that *E_o_* and the ratio *E_i_*/*E_o_* on the LHS and RHS of Equation (9), respectively, do not exist; the correct expression is indicated by Equation (12).

### 2.3. Tri-Layered Concentric Rods

**Figure 3:**
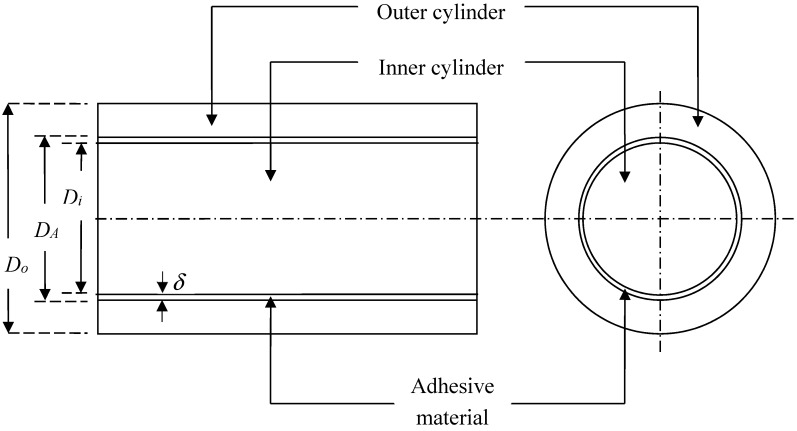
Side (**left**) and axial (**right**) views of a rod made from two concentric foams with opposite Poisson’s ratio signs, in which the adhesive material is assumed to take on the shape of a thin cylindrical shell.

Figure 3 shows the adhesive layer being the interlayer, thereby extending the bi-layered concentric rod into a more realistic tri-layered concentric rod with the thickness of the adhesive layer *δ* being

(15) $$\delta =\frac{D_{A}-D_{i}}{2}.$$

As in the case of a bi-layered concentric rod, the angular twist for a tri-layered rod is common

(16) $$\varphi_{i}=\varphi_{A}=\varphi_{o}$$

while the torsional load is carried by all three layers

(17) $$T=T_{i}+T_{A}+T_{o}.$$

Proceeding similarly as in the case of a bi-layered concentric rod, the effective Poisson’s ratio for a tri-layered concentric rod under torsional loading mode is

(18) $$v_{Tri-layer}={\left(\frac{64TL}{D_{o}^{4}E_{0}\pi \varphi }\right)}^{-1}-1$$

with

(19) $$\frac{64TL}{D_{o}{}^{4}E_{o}\pi \varphi }=\frac{\frac{E_{i}}{E_{o}}{\left(\frac{D_{i}}{D_{o}}\right)}^{4}}{1+v_{i}}+\frac{\frac{E_{A}}{E_{o}}\left[{\left(\frac{D_{A}}{D_{o}}\right)}^{4}-{\left(\frac{D_{i}}{D_{o}}\right)}^{4}\right]}{1+v_{A}}+\frac{1-{\left(\frac{D_{A}}{D_{o}}\right)}^{4}}{1+v_{o}}$$

where

(20) $${\left(\frac{D_{A}}{D_{o}}\right)}^{4}-{\left(\frac{D_{i}}{D_{o}}\right)}^{4}=8\frac{\delta }{D_{o}}{\left(\frac{D_{i}}{D_{o}}\right)}^{3}+24{\left(\frac{\delta }{D_{o}}\right)}^{2}{\left(\frac{D_{i}}{D_{o}}\right)}^{2}+32{\left(\frac{\delta }{D_{o}}\right)}^{3}\frac{D_{i}}{D_{o}}+16{\left(\frac{\delta }{D_{o}}\right)}^{4}$$

and

(21) $$1-{\left(\frac{D_{A}}{D_{o}}\right)}^{4}=1-{\left(\frac{D_{i}}{D_{o}}\right)}^{4}-8\frac{\delta }{D_{o}}{\left(\frac{D_{i}}{D_{o}}\right)}^{3}-24{\left(\frac{\delta }{D_{o}}\right)}^{2}{\left(\frac{D_{i}}{D_{o}}\right)}^{2}-32{\left(\frac{\delta }{D_{o}}\right)}^{3}\frac{D_{i}}{D_{o}}-16{\left(\frac{\delta }{D_{o}}\right)}^{4}.$$

It can be seen from Equations (20) and (21) that as *δ*→0 or *δ* << *D_o_*, Equation (19) reduces to Equation (9). If the adhesive layer is very small in comparison to other radial dimensions and the adhesive modulus is in the same order as that of the foam material, then the following simplifications of

(22) $${\left(\frac{D_{A}}{D_{o}}\right)}^{4}-{\left(\frac{D_{i}}{D_{o}}\right)}^{4}\approx 8\frac{\delta }{D_{o}}{\left(\frac{D_{i}}{D_{o}}\right)}^{3}$$

and

(23) $$1-{\left(\frac{D_{A}}{D_{o}}\right)}^{4}\approx 1-{\left(\frac{D_{i}}{D_{o}}\right)}^{4}$$

are valid for Equations (20) and (21), respectively. A direct consequence of this simplification is that Equation (19) resembles Equation (9); that is,

(24) $$\frac{64TL}{D_{o}{}^{4}E_{o}\pi \varphi }=\frac{\frac{E_{i}}{E_{o}}{\left(\frac{D_{i}}{D_{o}}\right)}^{4}}{1+v_{i}}+\frac{8\frac{E_{A}}{E_{o}}\frac{\delta }{D_{o}}{\left(\frac{D_{i}}{D_{o}}\right)}^{3}}{1+v_{A}}+\frac{1-{\left(\frac{D_{i}}{D_{o}}\right)}^{4}}{1+v_{o}}$$

such that the influence from the adhesive material is confined to only one term, that is, on the second term on the RHS of Equation (24). Here, the relative modulus of the adhesive-to-outer foam material *E_A_*/*E_o_* and the relative adhesive thickness *δ*/*D_o_* play equal importance. The simplification suggested in Equation (22), however, is no longer valid when the relative modulus of the adhesive material *E_A_*/*E_o_* is several orders higher. Hence, the retention of Equation (20) with the use of Equation (23) provides balanced simplification and accuracy. Without diminishing the adhesive layer, the tri-layered concentric rods reduce to bi-layered ones under the following special cases: (a) *D_i_* = 0, in which the adhesive layer takes the place of the inner core in the form of a very slim reinforcement rod, and (b) *D_i_* ≈ *D_o_* with a very thin *δ* in which the adhesive layer takes the place of the outer shell in the form of a very thin tube. It can be shown that as *D_i_*→0, Equation (24) reduces to

(25) $$\frac{64TL}{D_{o}{}^{4}E_{o}\pi \varphi }\approx \frac{1}{1+v_{o}}+\frac{8\frac{E_{A}}{E_{o}}\frac{\delta }{D_{o}}{\left(\frac{D_{i}}{D_{o}}\right)}^{3}}{1+v_{A}}.$$

Likewise, Equation (24) simplifies to

(26) $$\frac{64TL}{D_{o}{}^{4}E_{i}\pi \varphi }\approx \frac{1}{1+v_{i}}+\frac{8\frac{E_{A}}{E_{i}}\frac{\delta }{D_{o}}}{1+v_{A}}$$

as *D_i_→D_o_*. It can be observed that the limiting conditions for the tri-layered rod as specified in Equations (25) and (26) closely resemble those of the bi-layered cases of Equations (10) and (12), respectively. In the results section, curves of the effective Poisson’s ratio for the tri-layered rods are plotted against *D_i_*/*D_o_* for the range 0 ≤ *D_i_*/*D_o_* ≤ 1 in which both extreme cases *D_i_* = 0 and *D_i_* = *D_o_* are valid under the earlier imposed condition that *δ* << *D_o_*.

## 3. Results and Discussion

The influence of the intermediate layer on the overall Poisson’s ratio of a rod made from foams of opposing Poisson’s ratio signs can be observed by considering the adhesive layer’s elastic and geometrical properties. Specifically, these properties are (a) the elastic properties in terms of the adhesive modulus relative to that of the foam *E_A_*/*E_Foam_* and the adhesive Poisson’s ratio *v_A_*, as well as the (b) geometrical properties in terms of the adhesive thickness relative to the rod diameter *δ*/*D_o_* and the adhesive diameter relative to that of the rod *D_i_*/*D_o_*.

**Figure 4:**
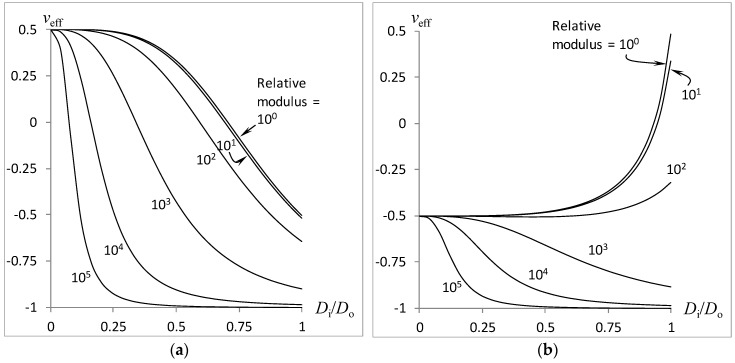
Effect of relative adhesive modulus on the compound rod auxeticity: (**a**) Auxetic core; (**b**) Auxetic shell.

**Figure 5:**
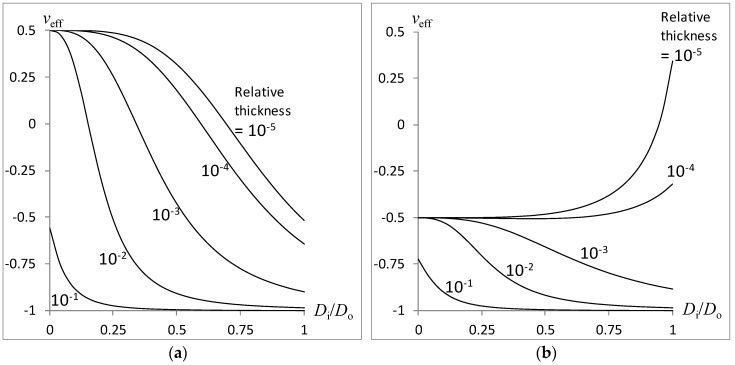
Effect of relative adhesive thickness on the compound rod auxeticity: (**a**) Auxetic core; (**b**) Auxetic shell.

**Figure 6:**
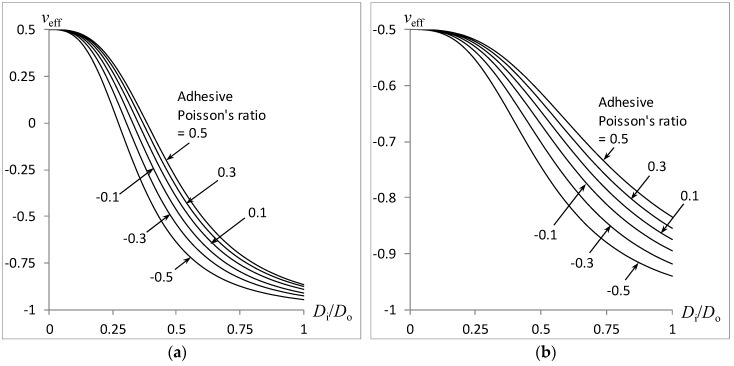
Effect of adhesive Poisson’s ratio on the compound rod auxeticity: (**a**) Auxetic core; (**b**) Auxetic shell.

In the plotted results of the effective Poisson’s ratio in Figure 4, Figure 5 and Figure 6, we adopt the concentrically tri-layered cylinder using Equations (18)–(20) and (23) for the case of an equal inner and outer foam modulus:

(27) $$E_{i}=E_{o}=E_{Foam},$$

and equal Poisson’s ratio magnitudes for the inner and outer foams

(28) $$\pm v_{i}=\mp v_{o}=0.5.$$

The effect of the relative moduli ratio *E_A_*/*E_Foam_* on the variation of the combined rod’s effective Poison’s ratio *v_eff_* with the inner-to-outer cylinder diameters *D_i_*/*D_o_* is plotted in Figure 4 with relative adhesive thickness at *δ*/*D_o_* = 0.001, Poisson’s ratio of solidified adhesive at *v_A_* = 0, and relative adhesive modulus at *E_A_*/*E_Foam_* = 10*^n^* for *n* = 0, 1, 2, 3, 4, 5. Figure 4a and Figure 4b refer to the auxetic core (−*v_i_* = *v_o_* = 0.5) and auxetic shell (*v_i_* = −*v_o_* = 0.5), respectively. As expected, the rod auxeticity increases (or the overall Poisson’s ratio decreases) with the relative size of the auxetic core, as shown in Figure 4a. However, an unexpected trend is observed whereby the overall Poisson’s ratio approaches −1 as the inner-to-outer diameter approaches 1, although none of the material components possess a Poisson’s ratio lower than −0.5. Plotted results also reveal that the rod auxeticity increases with the use of a higher adhesive modulus. When the position of the auxetic and conventional parts are swopped, only the case of moderate relative adhesive modulus gives an intuitive trend, that is, increasing Poisson’s ratio with increasing conventional inner cylinder size. However, the trend reverses for an extremely large adhesive modulus. This may well be due to the high torsional stiffness that translates into a high ratio, which is associated with auxeticity.

The effect of relative moduli thickness (*δ*/*D_o_*) on the variation of the combined rod’s effective Poison’s ratio *v_eff_* with the inner-to-outer cylinder diameters *D_i_*/*D_o_* is plotted in Figure 5 with relative adhesive modulus at *E_A_*/*E_Foam_* = 1000, Poisson’s ratio of solidified adhesive at *v_A_* = 0, and relative adhesive thickness at *δ*/*D_o_* = 10*^n^* for *n* = −1, −2, −3, −4, −5. Figure 5a,b correspond to the use of an auxetic core (−*v_i_* = *v_o_* = 0.5) and auxetic shell (*v_i_* = −*v_o_* = 0.5), respectively. The trends obtained in Figure 5 are somewhat similar to those of Figure 4, signifying an almost similar effect of adhesive thickness with adhesive modulus. The similarity is attributed to the increasing stiffness contributed by the intermediate layer’s increasing modulus and increasing thickness.

The effect of the adhesive’s Poisson’s ratio *v_A_* on the variation of the combined rod’s effective Poison’s ratio *v_eff_* with the inner-to-outer cylinder diameters *D_i_*/*D_o_* is plotted in Figure 6 with a relative adhesive modulus at *E_A_*/*E_Foam_* = 1000, relative adhesive thickness at *δ*/*D_o_* = 0.001, and adhesive Poisson’s ratio at *v_A_* = ±0.1, ±0.3, ±0.5. Figure 6a,b correspond to the use of an auxetic core (−*v_i_* = *v_o_* = 0.5) and auxetic shell (*v_i_* = −*v_o_* = 0.5), respectively. As with Figure 4 and Figure 5, a drop in the overall Poisson’s ratio towards −1 is obtained with increasing adhesive ring diameter. As expected, the overall Poisson ratio is influenced by the Poisson’s ratio of the adhesive material, thereby causing an upward or downward shift in the overall Poisson’s ratio arising from the positive or negative sign of the adhesive material’s Poisson’s ratio.

The current findings complement the results of Maksymuk and Shcherbina [6], which conclude that at a constant total thickness of the adhesive block the value of the tangential contact stresses can be regulated (by changing the geometric and physical-mechanical parameters of the layers and adhesive interlayer) in order to increase the rigidity characteristics of composite multilayer cylindrical shells in torsion. While the usual or direct approach for obtaining the effective Poisson’s ratio attempts to apply an axial load on the concentric rod such that the arising interfacial surface gap mismatch is removed by means of geometrical compatibility, the absence of radial change in the torsion of concentric rods circumvents the need for bridging the gap. The advantage of this simplicity, however, renders the current model inaccurate when considering combinations of foam materials with significant differences in their Young’s modulus. Specifically, the imposition of Equations (6) and (16) for the bi-layered and tri-layered rods, respectively, implies that the inner and outer foams must rotate by equal amounts of angle but this is not the case when the torsional stiffness of the inner core and outer shell are significantly different; in addition to *φ_i_* ≠ *φ_o_*, an interfacial shear stress acting in the circumferential direction arises. Nevertheless, an accuracy of ±10% is achieved; that is, *φ_i_* ≈ *φ_o_* for the case of auxetic core (*v_i_* = −0.5) and auxetic shell (*v_o_* = −0.5) when 10/11 ≤ *E_i_*/*E_o_* ≤ 10/9 and 30/31 ≤ *E_i_*/*E_o_* ≤ 30/29, respectively.

So far the choice of Poisson’s ratio of foams has been limited to *v* = ±0.5. For other auxetic-conventional combinations within this range, one may expect the following combination of factors:

(a) less pronounced effective Poisson’s ratio if the magnitudes are equal and less than 0.5,
(b) the outer shell plays a larger role than the inner core in influencing the effective Poisson’s ratio under bending and especially torsion, but is directly proportional to the cross-sectional area for the axial load,
(c) the foam with a larger Poisson’s ratio magnitude will exert a greater effect on the sign of the effective Poisson’s ratio.

## 4. Conclusions

The effect of adhesive properties on the overall auxeticity of a rod consisting of two concentrically aligned cylindrical isotropic foams with Poisson’s ratio of opposite signs—under torsional loading mode—has been investigated in this paper. Using the mechanics of materials approach, an indirect way for inferring the Poisson’s ratio of a concentrically multi-layered rod was obtained. Results show that the following factors increase the auxeticity of the rod under consideration: (a) adhesive modulus, (b) adhesive Poisson’s ratio, (c) adhesive thickness, and (d) adhesive radius. The plotted results also suggest that, even with each component possessing a Poisson’s ratio not lower than −0.5, the overall Poisson’s ratio within the framework of torsional loading approaches −1 as the outer shell thins. An understanding of the effect of adhesive elastic and geometrical properties of a rod made from concentrically aligned auxetic and conventional foams is useful for designing such rods whenever an adhesive with a large modulus is used for attaching both foams. Further refinements to the current model to cater for considerable differences in the inner and outer foams’ Young’s modulus is suggested for future work.
